# Comparison of creatinine- and cystatin C–based definitions of acute kidney injury in neonates with congenital diaphragmatic hernia

**DOI:** 10.1007/s00467-026-07156-2

**Published:** 2026-01-25

**Authors:** Judith Leyens, Jana Gerschlauer, Christoph Berg, Brigitte Strizek, Andreas Mueller, Florian Kipfmueller, Lukas Schroeder

**Affiliations:** 1 Department of Neonatology and Pediatric Intensive Care Medicine, University Children’s Hospital Bonn, Venusberg-Campus 1, 53127 Bonn, Germany; 2 Division of Congenital Malformations, Center for Rare Diseases Bonn, University Hospital Bonn, Bonn, Germany; 3 Division of Neonatology, Department of Pediatrics, BC Women’s and Children’s Hospital, University of British Columbia, Vancouver, Canada; 4 Department of Obstetrics and Prenatal Medicine, University Hospital Bonn, Bonn, Germany; 5 Department of Obstetrics and Prenatal Medicine, University Hospital Cologne, Cologne, Germany; 6 Department of Neonatology and Pediatric Intensive Care Medicine, University Children’s Hospital Mannheim, Mannheim, Germany

**Keywords:** Acute kidney injury, Congenital diaphragmatic hernia, Creatinine, Cystatin C

## Abstract

**Background:**

Acute kidney injury (AKI) is a frequent complication in critically ill neonates and is associated with adverse outcomes. Infants with congenital diaphragmatic hernia (CDH) are particularly vulnerable due to pulmonary hypoplasia, hemodynamic instability, and exposure to nephrotoxic agents.

**Methods:**

We retrospectively analyzed 193 neonates with CDH treated at a tertiary referral center (2012–2021). AKI was graded using modified pediatric RIFLE (pRIFLE), neonatal KDIGO (nKDIGO), and CysC-based neonatal AKI (CyNA) criteria, disregarding the urine output criteria. Clinical variables included CDH severity, extracorporeal membrane oxygenation (ECMO) use, and sepsis. Associations with AKI were examined using multivariable logistic regression.

**Results:**

AKI incidence varied significantly by definition (CyNA 82% > pRIFLE 78% > nKDIGO 56%; CyNA vs. pRIFLE/nKDIGO *p* = 0.028/*p* = 0.010). Moderate AKI was detected in 61% (CyNA), 38% (pRIFLE), and 26% (nKDIGO) of the cases. Severe AKI was detected in 42% (pRIFLE), 30% (nKDIGO), and 20% (CyNA) of the cases. Severe CyNA was significantly associated with in-hospital mortality (*p* = 0.003). The severity of AKI was associated with a stepwise increase in mortality (mild/moderate vs. severe, *p* < 0.01), as well as prolonged hospitalization, kidney replacement therapy, and hypertension at discharge. Independent risk factors for AKI included lower observed-to-expected lung-to-head ratio, sepsis, and ECMO.

**Conclusions:**

AKI is a frequent and clinically important complication in neonates with CDH and is strongly associated with overall disease severity, incidence of sepsis, and ECMO. Neonates with severe AKI have a higher mortality. CysC might serve as an additional biomarker for the detection of neonates with CDH and fatal outcome. Future studies should assess whether integrating both serum creatinine and CysC into AKI monitoring improves the early detection of neonates with CDH at risk of acute and chronic kidney disease.

**Graphical abstract:**

A higher resolution version of the Graphical abstract is available as Supplementary information.

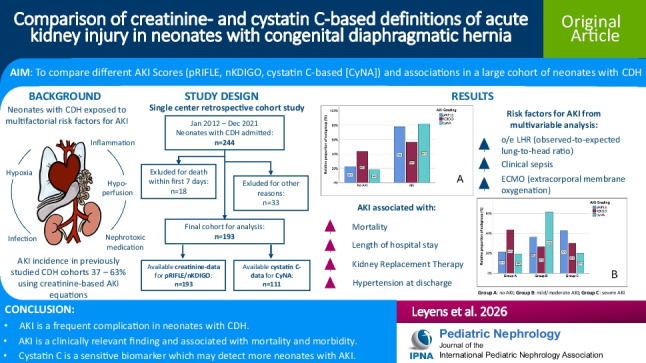

**Supplementary Information:**

The online version contains supplementary material available at 10.1007/s00467-026-07156-2.

## Introduction

Neonatal acute kidney injury (AKI) has gained increasing recognition in recent years as a significant complication impacting short- and long-term morbidity and mortality. Various scoring and grading systems for neonatal AKI have been established through large multicenter cohorts [1, 2]. Historically, AKI has been defined by established scores using a rise in serum creatinine (SCr) or a decreased urine output as surrogate markers of impaired kidney function [3]. In the pediatric population, established criteria include the pediatric Risk Injury Failure and End-Stage (pRIFLE), the AKI-Network (AKIN), and the modified neonatal Kidney Disease Improving Global Outcome (nKDIGO) score [4–6]. To date, there is no evidence or consensus guideline favoring the use of a certain score over another in the neonatal population, but the nKDIGO appears to be most commonly used [7, 8].

However, both SCr and urine output are influenced by multiple factors and have limitations, especially in neonates: an increase in SCr can be delayed after a kidney injury, serum levels correlate with skeletal muscle mass and are gestational age (GA)-dependent, and as SCr crosses the feto-placental barrier, neonatal SCr levels primarily reflect maternal values in the first 5 days of life [1, 9, 10]. Therefore, other biomarkers, such as the cysteine protease inhibitor cystatin C (CysC), have been investigated as diagnostic criteria for AKI. Several factors make CysC an ideal surrogate marker for kidney dysfunction: it is produced by all nucleated cells, freely filtered by the glomeruli, and almost entirely reabsorbed and catabolized in the proximal tubule without kidney secretion [11]. CysC is independent of GA or inflammation, and neonatal levels are independent of maternal levels [12]. Even small reductions in glomerular filtration rate cause a significant increase in CysC, which makes it an ideal early marker for AKI [13]. Xu et al. [10] have proposed a CysC-derived neonatal AKI (CyNA) definition and grading criteria, which may identify 5.5 times greater neonates with AKI. It is important to note that SCr and CysC should not be regarded as direct kidney injury biomarkers. Rather, they are markers of kidney dysfunction, as they are not actively released in response to kidney injury like more sensitive biomarkers such as urinary neutrophil gelatinase–associated lipocalin (NGAL). Instead, a reduction in glomerular filtration rate leads to a secondary and delayed increase in SCr and CysC.

Incidence of neonatal AKI in preterm and term neonates using traditional AKI criteria ranges from 18–40%, but may be higher in neonates with additional risk factors, such as congenital anomalies with hemodynamic or oxygenation impairment [2, 14, 15]. Neonates with congenital diaphragmatic hernia (CDH) are impacted by pulmonary hypoplasia of varying degrees, which puts them at risk for pulmonary hypertension, cardiac dysfunction, and systemic hemodynamic mal-perfusion and hypoxia [16]. Recent studies have also shown that neonates with CDH have a high risk of AKI, which significantly increases their morbidity and mortality [17–23]. Reported incidences vary significantly from 37 to 63% depending on the composition of the evaluated cohorts and AKI criteria used. Several additional risk factors, such as extracorporeal membrane oxygenation (ECMO), nephrotoxic medication such as loop diuretics or antibiotics, as well as perioperative complications during surgical hernia repair, have been shown to increase the risk of AKI in these infants even further [23].

To date, there are no studies that investigate the use of CysC to detect AKI in neonates with CDH and follow the longitudinal course of biomarkers evaluating kidney dysfunction. The aim of this study is to compare different AKI scores (modified pRIFLE, nKDIGO, and CyNA) and evaluate the longitudinal course of AKI during the in-hospital stay in a large cohort of almost 200 neonates with CDH.

## Material and methods

### Study design and patient selection

**Fig. 1:**
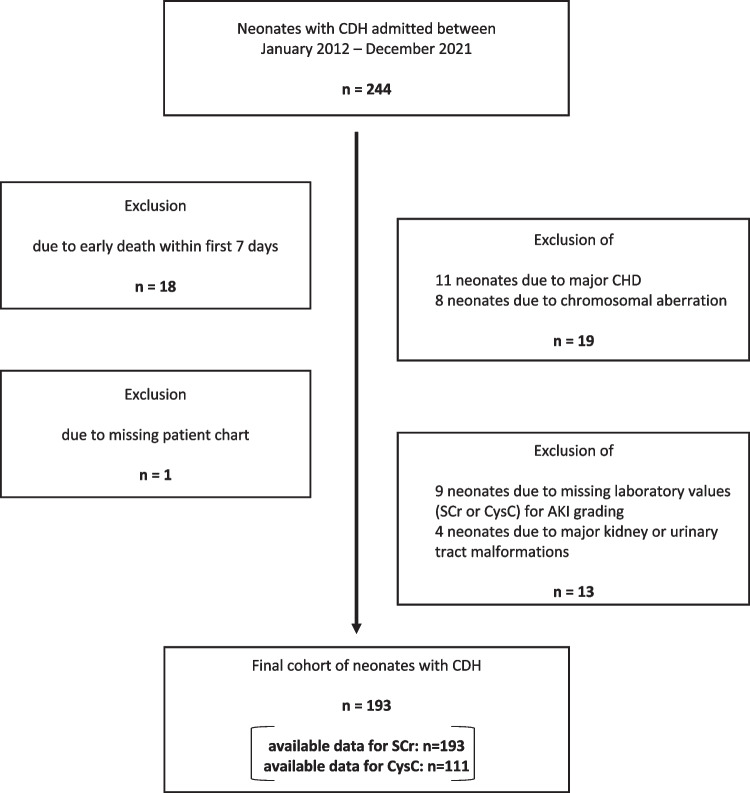
Flowchart of the retrospective patient selection and the application of exclusion criteria. AKI, acute kidney injury; CDH, congenital diaphragmatic hernia; CHD, congenital heart disease; CysC: cystatin C; SCr: serum creatinine

We conducted a retrospective analysis of neonates with CDH treated at our quaternary referral center in the 10-year period between January 01, 2012, and December 31, 2021. Outborn and inborn neonates with CDH were eligible for study inclusion. Exclusion criteria were as follows: palliative care after birth or death in the first week of life, major congenital heart disease (except patent ductus arteriosus, atrial septal defect, or non-significant ventricular septal defect), chromosomal anomalies or genetic syndromes, malformations of the urinary tract and kidneys, missing SCr data for AKI grading. Laboratory values of CysC were optional. A flow-chart of patient in- and exclusion is illustrated in Fig. 1.

#### Ethics approval

This study was approved by the local ethics committee of the Medical Center of the University of Bonn (local running number 030/22). Written informed consent was waived due to the retrospective design of the study. The methods used for the clinical research were performed in accordance with the STROBE (strengthening the reporting of observational studies in epidemiology) guidelines and in accordance with the Declaration of Helsinki.

### Data collection

Data were collected from paper charts for patients born before December 2019 and from the electronic medical records thereafter (ICM‐PDMS, Draeger Medical Germany GmbH, Lübeck, Germany; Neodat, Paedsoft, Tübingen, Germany; Orbis, Dedalus Healthcare GmbH, Bonn, Germany). Collected demographic data included: sex, GA in weeks, birth weight (BW), length of hospital stay (LOS), and in-hospital mortality. Data defining CDH severity included: CDH location (right/left), intrathoracic liver herniation, observed-to-expected lung-to-head ratio (o/e LHR %), antenatal fetal endoluminal tracheal occlusion (FETO), defect size according to the CDH study group (CDHSG) classification. Neonatal treatment data and risk factors for AKI included: need for ECMO or kidney replacement therapy (KRT), vancomycin and/or tobramycin administration, duration, and toxic plasma levels, use of umbilical arterial catheter, clinical sepsis (defined as clinical deterioration with fever > 38 °C, tachycardia > 200/min, capillary filling time > 2 s, which led to an intensification of antibiotic therapy), blood culture positive sepsis, and antihypertensive therapy at discharge.

### AKI biomarkers and scoring systems

**Fig. 2:**
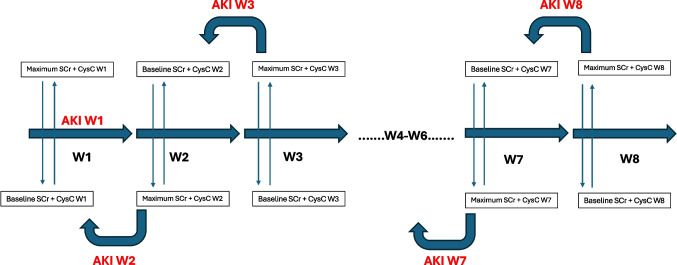
Procedure of the calculation of SCr and CysC baseline values from week 1 (W1) to week 8 (W8)

The blood biomarkers SCr and CysC, which evaluate kidney dysfunction but not urine output, were used for AKI definition. CysC and SCr were measured in the main laboratory, using an immunoassay for CysC (cobas® c703, Roche Diagnostics), with a sample volume from the micro-cup (including dead volume) of 52 µl, and SCr using VIS photometry (cobas® c703, Roche Diagnostics Ic703), with a sample volume from the micro-cup (including dead volume) of 60 µl. AKI biomarkers were screened until week 8 of life (W8). The lowest CysC within the first week of life (W1, days 1–7), and the lowest SCr within 48 h and the 7th day of life (days 3–7), were collected and defined as the baseline reference value (W1). SCr values in the first 2 days of life were not considered to ensure no interference from maternal SCr values after birth. Minimum and maximum CysC and SCr values were calculated weekly until W8. Baselines (weekly minimum value) for CysC and SCr were re-calculated weekly (W2, W3, etc.). To calculate the weekly AKI incidence, the maximum values for CysC and SCr were compared with the baseline value of the preceding week (see Fig. 2). To diagnose AKI, pRIFLE, nKDIGO, and CyNA were calculated as demonstrated in Table 1 [8, 23, 24]. For further analysis, neonates were then allocated to one of three groups depending on AKI severity: no AKI (Group A), mild to moderate AKI (Group B), and severe AKI (Group C), as demonstrated in Table 1. For ECMO patients, kidney dysfunction biomarkers were trended daily during their first 10 days on ECMO in relation to the secondary outcome of in-hospital mortality.

**Table 1:** Overview of the different AKI scoring criteria used in this study

Stage	AKI scoring	Group allocation
pRIFLE (modified according to Ryan et al. [5])		
Stage 0	No-risk: < 25% increase from baseline SCr	A
Stage 1	Risk: > 25% increase from baseline SCr	B
Stage 2	Injury: > 50% increase from baseline SCr	B
Stage 3	Failure: > 75% increase from baseline SCr	C
Stage 4	Loss of function: failure persisting > 4 weeks	C
Stage 5	End-stage: < 10% renal function for > 3 month	C
nKDIGO		
Stage 0	No change, or increase < 0.3 mg/dl, or < 1.5 times from baseline SCr	A
Stage 1	Increase in SCr ≥ 0.3 mg/dl within 48 h or ≥ 1.5–1.9 times from baseline SCr within 7 days	B
Stage 2	Increase in SCr ≥ 2.0–2.9 times from baseline SCr	C
Stage 3	Increase in SCr ≥ 3 times from baseline SCr, or SCr ≥ 2.5 mg/dl, or need for RRT	C
CyNA [12]		
Stage 0	CysC < 2.2 mg/dl, or ≤ 25% increase from baseline CysC	A
Stage 1	CysC ≥ 25% increase from baseline CysC, but < 2.2 mg/dl	B
Stage 2	CysC > 2.2 mg/dl	C

Based on AKI severity, neonates were then allocated to either group A (no AKI), group B (mild to moderate AKI), or group C (severe AKI) for further analysis. Baseline SCr and CysC in W1 are defined as the minimum SCr within 48 h–7 days of life and the minimum CysC measured within the first week of life. The baseline values of W2–W8 are the minimum values of each week

*AKI* acute kidney injury, *CyNA* cystatin C-related criteria for neonatal AKI, *CysC* cystatin C, *nKDIGO* modified neonatal Kidney Disease Improving Global Outcome, *pRIFLE* pediatric risk injury failure and end-stage, *RRT* renal replacement therapy, *SCr* serum creatinine

#### CDH management and KRT

Local CDH management was conducted as previously published [25, 26]. Treatment was aligned with the recommendations of the CDH Euro Consortium group [27], and consisted of the following key strategies: (I) lung-protective ventilation, (II) treatment of PH and CD, (III) use of ECMO for oxygenation failure, and (IV) delayed surgical repair. The primary mode for ECMO in our institution is veno-venous. If required, significant fluid overload and edema were treated with the following diuretics as deemed appropriate by the most responsible attending physician: (I) furosemide (continuous infusion 1–10 mg/kg/day), (II) ethacrynic acid (intermittent infusion 1–3 mg/kg/day), or (III) chlorothiazide (intermittent infusion 10–40 mg/kg/day). Indications for KRT were (1) significant capillary leak and fluid overload unresponsive to conservative treatment, (2) pRIFLE/nKDIGO stage ≧ C, (3) blood urea nitrogen (BUN) levels ≥ 250 mg/dl, or (4) anuria for > 24–48 h. KRT was either implemented into the ECMO circuit, if applicable, or via a separate central Shaldon dialysis catheter. Minimum weight for ECMO and/or KRT eligibility was 1800 g.

#### Outcome measures, sample size estimation, and statistical analysis

The diagnosis of any AKI (mild to severe, group B/C) according to pRIFLE, nKDIGO, or CyNA criteria was defined as the primary outcome. The following parameters were defined as secondary outcomes: (a) stage of AKI, (b) LOS, (c) survival to discharge, and (d) need for KRT. For the power calculation [28], we used the following assumptions: the allocation rate for two-group testing (no AKI versus AKI: N2/N1 = 0.5), an effect size of *d* = 0.5, a power of 0.9, and an alpha error of 0.05. The adequate sample sizes for two-group testing were calculated as group 1 (AKI) 109 patients and group 2 (no AKI) 55 patients, when the incidence (AKI) of ~ 60% was estimated.

Normally distributed data are displayed as mean with standard deviation (± SD), and non-normally distributed data are displayed as median with inter-quartile range (IQR). Wilcoxon, Mann–Whitney *U* test, or ANOVA for repeated measurements, with Holm–Bonferroni correction, was used to compare continuous variables, as appropriate. Pearson’s Chi^2^ test and Fisher’s exact test were applied for categorical variables, as appropriate. Kaplan–Meier plots were conducted for the calculation of the cumulative survival during the in-hospital stay. A binary regression model was used to adjust for influencing covariables. Metric variables included in the model were normalized with the natural logarithm (ln) using the Box–Tidwell method. A *p*-value of < 0.05 was considered significant. The statistical analysis was performed using statistical software (IBM SPSS Statistics for Windows, Version 27.0., IBM Corp, Armonk, NY).

## Results

Of 244 neonates with CDH, 51 were excluded, resulting in a final cohort of 193 neonates for AKI study inclusion **(**Fig. 1). Baseline demographics and treatment data are demonstrated in Table 2. Most neonates were born at term (mean GA, 38 weeks) with a BW appropriate for GA (median, 3 kg). Most were male (57%), had a left-sided CDH (84%), and had an intrathoracic liver herniation (62%). The median o/e LHR was 40%, and 30 infants (16%) had antenatal FETO treatment. Intraoperative CDHSG defect size classification was available for 147 neonates (76%). Of these, 57% (84 neonates) had a higher-risk Type C/D lesion. Patch repair was required in 112 infants (58%). ECMO was used in 80 infants (42%), and KRT was required in 13 infants (7%). The median LOS was 44 days, and the overall mortality was 18.1%. Culture-positive sepsis occurred in 24 infants (12%). Vancomycin was more commonly used than tobramycin (70% vs. 31%), and toxic vancomycin levels were documented in 15%. No toxic tobramycin levels were documented. Antihypertensive medication at discharge was present in 21% of infants, and 2% required dual antihypertensive therapy.

**Table 2:** Overview of baseline demographics, CDH severity and treatment data, and risk factors during treatment for AKI

	Total Cohort	pRFILE (AKI)	nKDIGO (AKI)	CyNA (AKI)	pRIFLE (*p*-value)	nKDIGO (*p*-value)	CyNA (*p*-value)
**Baseline demographics**						AKI vs. no AKI	
Included patients, *n* (%)	193	193	193	111			
AKI incidence, *n* (%)		150 (78)	109 (56)	91 (82)			
Female sex, *n* (%)	83 (43)	83 (55)	56 (51)	55 (60)	0.485	0.080	0.99
GA, weeks (IQR)	38 (37/39)	38 (35/39)	38 (37/39)	38 (36/39)	0.956	0.843	0.013
BW, kg (IQR)	3 (2.5/3.4)	2.9 (2.5/3.3)	2.9 (2.5/3.3)	3 (2.5/3.4)	0.159	0.229	0.016
LOS, *d* (IQR)	44 (29/74)	50 (30/78)	58 (36/81)	59 (33/88)	< 0.001	< 0.001	0.084
In-hospital mortality, *n* (%)	35 (18)	33 (22)	28 (26)	32 (35)	0.007	0.002	0.032
**CDH-severity**							
FETO, *n* (%)	30 (16)	27 (18)	21 (19)	22 (24)	0.096	0.114	0.234
o/e LHR, %	40 (32/48)	38 (38/46)	36 (30/44)	35 (30/43)	< 0.001	< 0.001	0.001
CDH left-sided, *n* (%)	162 (84)	127 (85)	94 (86)	76 (83)	0.857	0.911	0.296
Liver-herniation, *n* (%)	119 (62)	100 (67)	78 (72)	71 (78)	0.012	0.002	0.005
Defect size (CDHSG)					0.002	0.002	0.010
Defect size A, *n* (%)	14 (7)	10 (7)	6 (6)	3 (3)	-	-	-
Defect size B, *n* (%)	49 (25)	33 (22)	20 (18)	14 (15)	-	-	-
Defect size C, *n* (%)	58 (30)	47 (31)	35 (32)	34 37)	-	-	-
Defect size D, *n* (%)	26 (14)	25 (17)	21 (19)	20 (22)	-	-	-
Defect size unknown, *n* (%)		35 (23)	27 (25)	20 (22)			
CDH patch-repair, *n* (%)	112 (58)	97 (65)	79 (72)	69 (76)	< 0.001	< 0.001	0.078
**Treatment data and AKI risk factors**							
ECMO, *n* (%)	80 (42)	73 (49)	61 (56)	69 (76)	< 0.001	< 0.001	< 0.001
Clinical sepsis, *n* (%)	134 (70)	115 (77)	93 (85)	82 (90)	< 0.001	< 0.001	0.028
Culture-positive sepsis, *n* (%)	24 (12)	21 (14)	16 (15)	20 (22)	0.99	0.813	0.037
UAC, *n* (%)	42 (22)	31 (21)	25 (23)	19 (21)	0.531	0.726	0.99
AKI onset, week of life (IQR)	2 (1/2)	2 (1/2)	2 (1/2)	1 (1/2)			
KRT, *n* (%)	13 (7)	12 (8)	11 (10)	13 (14)	0.304	0.042	0.120
Vancomycin-administration, *n* (%)	135 (70)	114 (76)	93 (85)	81 (89)	0.001	< 0.001	0.142
Duration of vancomycin- administration, *d* (IQR)	16 (8/30)	19 (9/35)	20 (9/35)	20 (9/39)	0.010	0.038	0.977
Plasma level vancomycin > 20 µg/ml, *n* (%)	29 (15)	25 (17)	22 (20)	23 (25)	0.99	0.498	0.753
Tobramycin administration, *n* (%)	59 (31)	41 (27)	33 (30)	23 (25)	0.090	0.99	0.779
Duration of tobramycin administration, d (IQR)	5 (4/8)	5 (4/8)	5 (4/7)	4 (4/5)	0.69	0.534	0.864
Antihypertensive medication at discharge, *n* (%)	40 (21)	37 (25)	28 (26)	24 (26)	0.002	0.060	0.783
Dual antihypertensive treatment at discharge, *n* (%)	4 (2)	4 (3)	3 (3)	3 (3)	0.99	0.99	0.99

Data were compared using univariate analysis for neonates with and without AKI as defined by the individual neonatal AKI criteria pRIFLE, nKDIGO, and CyNA. Statistically significant *p*-values < 0.05 are highlighted in bold

*AKI* acute kidney injury, *BW* birth weight, *CDH* congenital diaphragmatic hernia, *CDHSC* congenital diaphragmatic hernia study scale, *CyNA* cystatin C-related criteria for neonatal AKI, *CysC* cystatin C, *ECMO* extracorporeal membrane oxygenation, *FETO* fetal endoluminal tracheal occlusion, *GA* gestational age, *LOS* length of hospital stay, *nKDIGO* modified neonatal Kidney Disease Improving Global Outcome, *OR* odds ratio, *pRIFLE* pediatric Risk Injury Failure and End-Stage, *KRT* kidney replacement therapy, *SCr* serum creatinine, *UAC* umbilical arterial catheter

### Incidence and severity of AKI

**Fig. 3:**
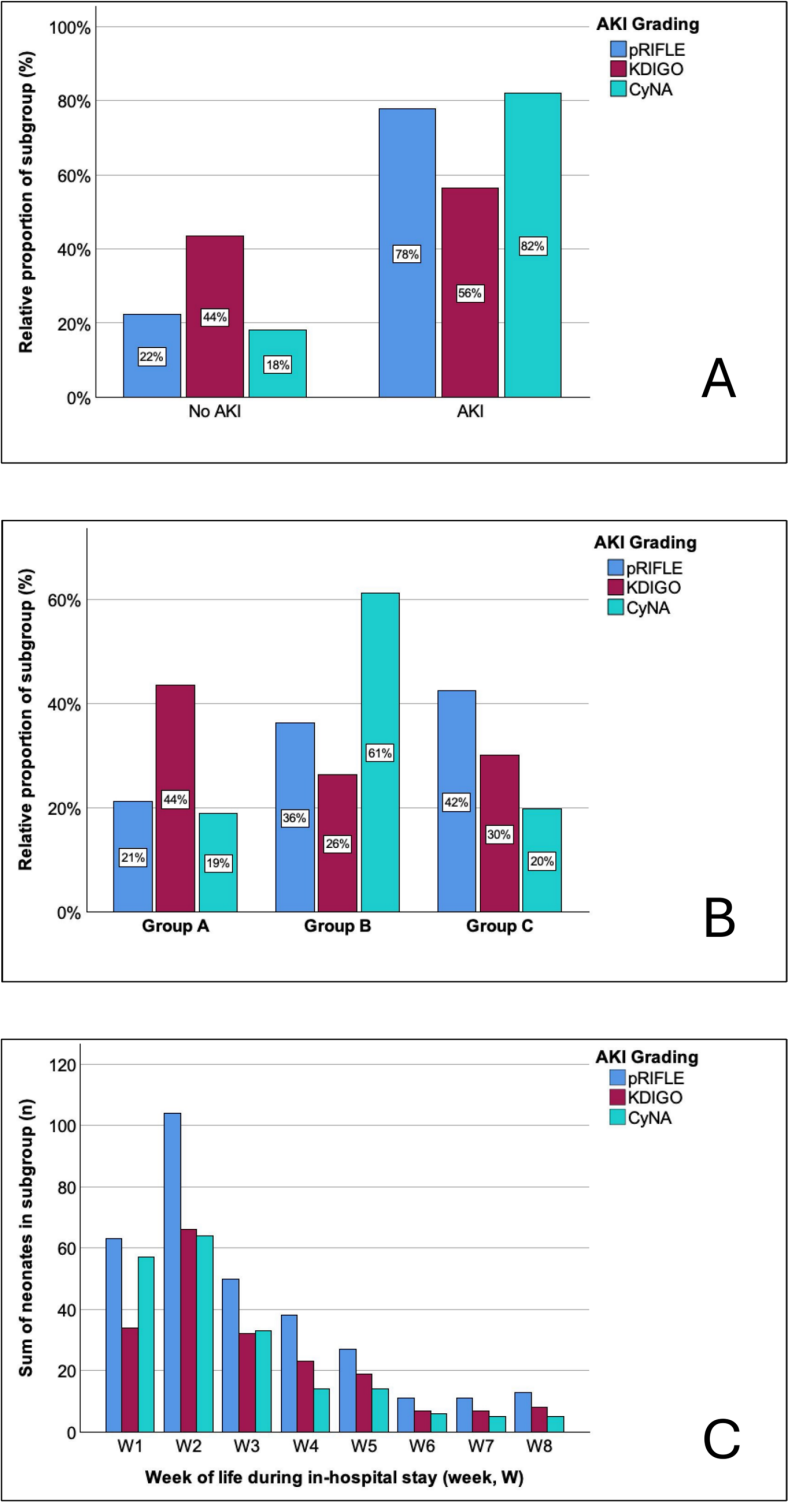
Demonstration of overall AKI incidence regarding to the different AKI diagnostic criteria (**A**), AKI severity score regarding the individual scoring systems (**B**), and timing of occurrence of AKI in weeks of life for the different AKI diagnostic criteria (**C**). AKI, acute kidney injury; CyNA, cystatin C-related criteria for neonatal AKI; nKDIGO, modified neonatal Kidney Disease Improving Global Outcome; OR, odds ratio; pRIFLE, pediatric risk injury failure and end-stage

The median number for CysC samples per patient was 2 (0/9) and 15 (9/22) for SCr during the in-hospital stay. SCr values to grade AKI as per nKDIGO and pRIFLE were available for 193 infants (100%), and CysC values to grade AKI as per CyNA were available for 111 infants (58%). Median baseline SCr was 0.41 (0.28/0.54), and median baseline CysC was 0.84 (0.72/1.0). Incidence of AKI ranged from 56 to 82% (CyNA 82% > pRIFLE 78% > nKDIGO 56%) (Fig. 3A). Moderate AKI was present in 26–61% (CyNA 61% > pRIFLE 36% > nKDIGO 26%) and severe AKI was present in 20–42% (pRIFLE 42% > nKDIGO 30% > CyNA 20%) (Fig. 3B). Most infants developed AKI during the second week of life, with a decrease in the AKI incidences between weeks three and six, and a stabilization of AKI incidences at a lower level between weeks six and eight (Fig. 3C). Mortality was significantly associated with increased AKI severity (*p* < 0.01): infants with no AKI only had a mortality rate of 1–4% (pRIFLE < CyNA < nKDIGO), which increased to 5–17% in neonates with mild to moderate AKI (pRIFLE < nKDIGO < CyNA), and 10–12% in neonates with severe AKI (CyNA < nKDIGO < pRIFLE).

### Univariate analysis

Neonates with AKI according to pRIFLE and nKDIGO had a lower o/e LHR (*p* < 0.001), more severe defect size (*p* = 0.002), a higher incidence of liver-herniation (*p* = 0.012, and *p* = 0.002, respectively), and a higher rate of patch repair (*p* < 0.001) in the univariate analysis (Table 2). They also experienced higher rates of clinical sepsis (*p* < 0.001), but not culture-positive sepsis, more frequent ECMO (*p* < 0.001) and KRT (p_nKDIGO_ = 0.042) use, and more frequent vancomycin administration (*p* = 0.001, and *p* < 0.001, respectively). Neonates with AKI required antihypertensive medication at discharge more frequently (*p* = 0.002, and *p* = 0.060, respectively), and they had a significantly longer LOS (*p* < 0.001) and higher mortality (*p* = 0.007, and *p* = 0.002, respectively). Univariate analysis for neonates with AKI defined by CyNA demonstrated slightly different results: neonates with CyNA-defined AKI had a significantly lower GA and BW (*p* = 0.013, and *p* = 0.016, respectively), but also had more ECMO use (*p* < 0.001), and signs of clinical sepsis (*p* = 0.028). In addition, these neonates had significantly higher rates of culture-proven sepsis (*p* = 0.037).

### Longitudinal kidney biomarker measurements

**Fig. 4:**
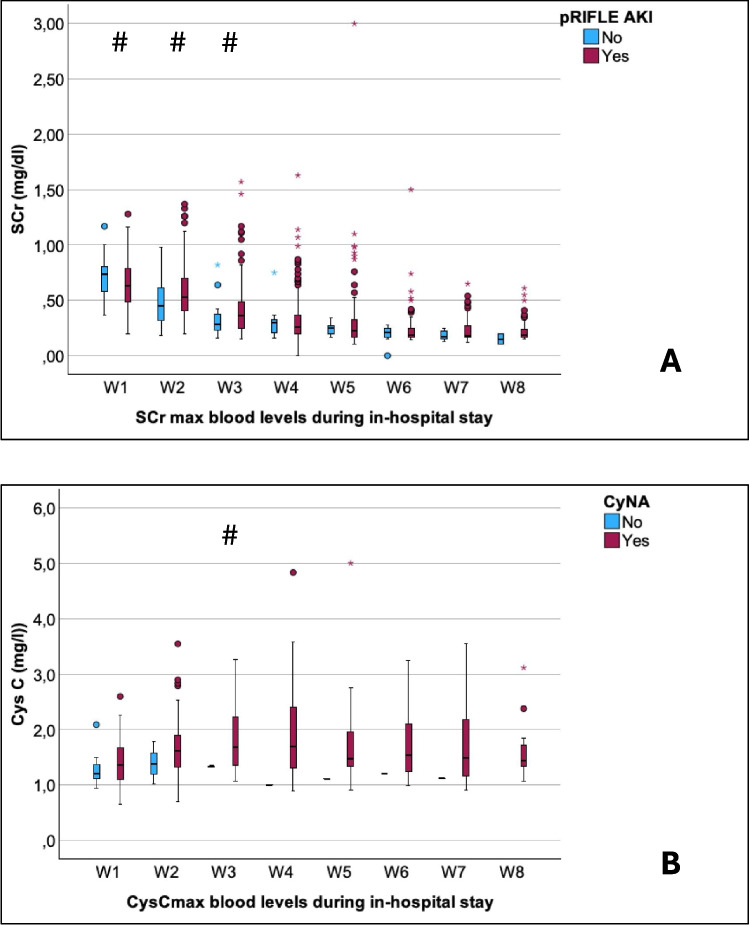
Weekly measurements of SCr_max_ (**A**) and CysC_max_ (**B**) are demonstrated in relation to evidence of AKI defined by pRIFLE. Significant differences between AKI and no-AKI are displayed with an asterisk in the figure. AKI, acute kidney injury; CysC, cystatin C; Max, maximum; SCr, serum creatinine; pRIFLE, pediatric risk injury failure and end-stage

**Fig. 5:**
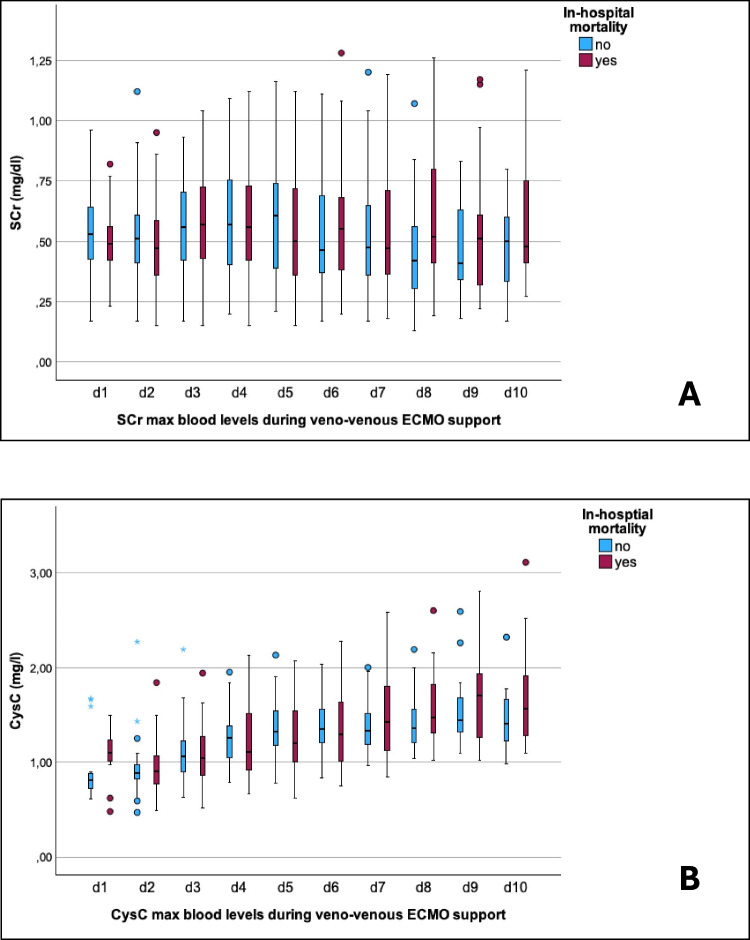
Kidney biomarkers SCr (**A**) and CysC (**B**) were trended daily in neonates on ECMO during their first 10 days of extracorporeal support in relation to the secondary outcome of in-hospital mortality. No significant differences between survivors and non-survivors were found. AKI, acute kidney injury; CysC, cystatin C; ECMO, extracorporeal membrane oxygenation; SCr, serum creatinine

Weekly measurements of SCr_max_ and CysC_max_ in relation to evidence of AKI defined by pRIFLE are illustrated in Fig. 4A–B. No significant differences were found for CysC_max_ at any point. SCr_max_ was significantly increased in neonates with AKI during the first three weeks. In our cohort, 49–76% of all neonates requiring ECMO developed AKI (see Table 2). Daily trends of biomarkers on ECMO showed no significant difference in CysC or SCr between survivors and non-survivors (Fig. 5A–C). CysC on the first day of ECMO tended to be higher in non-survivors, although without achieving statistical significance (*p* = 0.102).

### Binary regression model and Kaplan–Meier plot

Using binary logistic regression models, the following three pre- and postnatal factors were identified as independently associated with the diagnosis of AKI (Table 3): (I) o/e LHR (OR_nKDIGO_ = 0.21 (0.06/0.8) and OR_pRIFLE_ = 0.19 (0.04/0.9)), (II) clinical sepsis (OR_nKDIGO_ = 2.8 (1.1/7.1)), and (III) ECMO (OR_pRIFLE_ = 3.6 (1.2/10.2), OR_nKDIGO_ = 2.3 (1.0/5.1), OR_CyNA_ = 8.7 (2.4/32)).

**Table 3:** Binary logistic regression models to identify independent risk factors for the evolution of AKI defined by the three individual diagnostic criteria pRIFLE, nKDIGO, and CyNA in neonates with CDH

	Model 1 (pRIFLE)	Model 2 (nKDIGO)	Model 3 (CyNA)
Model quality	OR, 32; Cox *R*^2^, 0.176; Nagelkerke’s *R*^2^, 0.261	OR, 46; Cox *R*^2^, 0.239; Nagelkerke’s *R*^2^, 0.320	OR 27; Cox *R*^2^, 0.240; Nagelkerke’s *R*^2^, 0.383
	OR (95%CI)	OR (95%CI)	OR 95%CI
ECMO	3.6 (1.2/10.2)	2.3 (1.0/5.1)	8.7 (2.4/32)
Clinical sepsis	2.2 (0.8/5.9)	2.8 (1.1/7.1)	2.3 (0.5/9.9)
GA	2.3 (0.007/782)	4.1 (0.02/851)	0.001 (0/177)
o/e LHR	0.19 (0.04/0.9)	0.21 (0.06/0.8)	0.3 (0.03/2.5)
Vancomycin use	1.2 (0.4/3.2)	1.9 (0.7/5.0)	-

Statistically significant *p*-values < 0.05 are highlighted in bold. Data are presented as odds-ratio with 95% confidence interval

*AKI* acute kidney injury, *CDH* congenital diaphragmatic hernia, *CyNA* cystatin C-related criteria for neonatal AKI, *ECMO* extracorporeal membrane oxygenation, *GA* gestational age, *nKDIGO* modified neonatal Kidney Disease Improving Global Outcome, *o/e LHR* observed-to-expected lung-to-head ratio, *OR* odds ratio, *pRIFLE* pediatric Risk Injury Failure and End-Stage, *SCr* serum creatinine

**Fig. 6:**
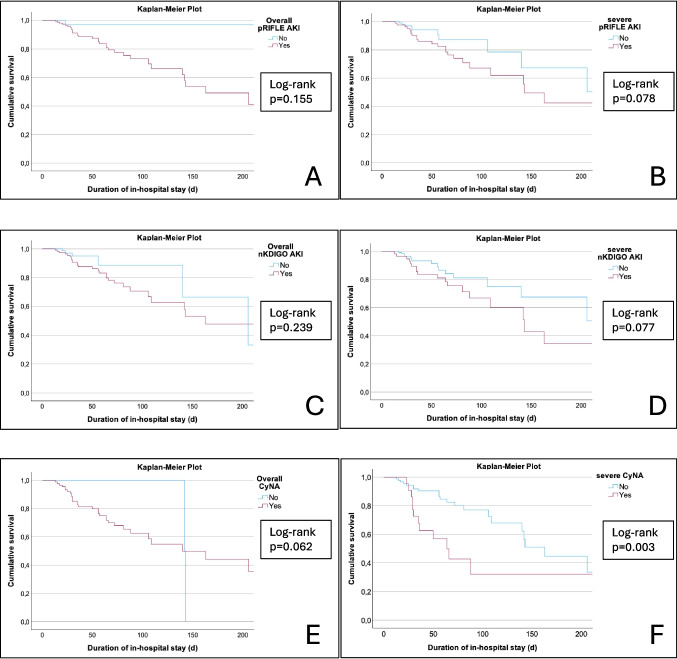
Kaplan–Meier plots depicting survival in relation to the primary outcome evidence of overall and severe AKI defined by pRIFLE (A/B), nKDIGO (C/D), CyNA (E/F). AKI, acute kidney injury; CyNA, cystatin C-related criteria for neonatal AKI; nKDIGO, modified neonatal Kidney Disease Improving Global Outcome; pRIFLE, pediatric risk injury failure and end-stage

The cumulative survival in relation to the primary outcome diagnosis of overall and severe AKI is demonstrated using Kaplan–Meier plots (Fig. 6A–E). Diagnosis of severe CyNA, but not overall CyNA, significantly discriminated between survivors and non-survivors (*p* = 0.003). In contrast, no statistically significant differences between survivors and non-survivors were observed in the Kaplan–Meier analysis for either overall or severe AKI when applying the pRIFLE or nKIDGO criteria.

## Discussion

Our study evaluates a large monocentric cohort of neonates with CDH for evidence of AKI and is the first to include data on CysC and CyNA-defined AKI. The main findings of our study are as follows: AKI has a high incidence in neonates with CDH, ranging from 56 to 82% (CyNA 82% > pRIFLE 78% > nKDIGO 56%). Most AKI cases were diagnosed using the CyNA criteria (82%), with notably more cases classified as moderate AKI (61%) compared with pRIFLE (36%) and nKDIGO (26%). In contrast, the highest proportion of severe AKI cases was identified using the pRIFLE criteria (42%), followed by nKDIGO (30%) and CyNA (20%). Severe AKI defined by CyNA criteria was significantly associated with in-hospital mortality. Infants with AKI had a significantly higher need for KRT, longer LOS, as well as higher mortality, and more commonly required antihypertensive medication at discharge. There is a gradually increased risk of mortality with increased AKI severity. Risk factors for AKI included low BW, lower o/e LHR, intrathoracic liver herniation, high-risk CDH defect size and patch repair, clinical sepsis, and vancomycin use. In the binary logistic regression models, clinical sepsis, o/e LHR, and ECMO were identified as independently associated with the evolution of AKI.

### Does CyNA outperform pRIFLE or KDIGO criteria?

While the inclusion of CysC-based CyNA criteria resulted in a higher overall incidence of AKI, our findings do not uniformly support a general superiority of CysC-based definitions over SCr-based criteria. Indeed, SCr-based AKI definitions showed stronger associations with length of hospital stay, and neither SCr- nor CysC-based definitions demonstrated a significant association with in-hospital mortality for overall AKI in the Kaplan–Meier plots. However, when focusing on severe AKI defined by CyNA, AKI was significantly associated with in-hospital mortality (log-rank test *p* = 0.003). In the multivariable regression models, CyNA showed a limited number of independent associations compared with SCr-based definitions, with ECMO emerging as the only significant covariate. These findings suggest that while CysC-based criteria do not consistently outperform creatinine-based definitions across all outcome measures, severe CyNA may identify a subgroup of neonates at particularly high risk of mortality. Overall, SCr and CysC appear to reflect different, complementary aspects of kidney dysfunction and disease severity, emphasizing the importance of combining both biomarkers.

CysC has some potential advantages over SCr, especially in the early neonatal or critically ill populations. In contrast to SCr, CysC is less affected by maternal factors, inflammation, or GA, which might make it an excellent marker of neonatal kidney dysfunction, especially in the early postnatal days when SCr measurements may still be reflective of maternal values [12, 29, 30]. The combination of both SCr and CysC may be most valuable, as it appears unlikely to miss AKI if both markers are within normal limits [10, 31]. One potential advantage of SCr is that it is readily available in some units for point-of-care testing (POCT). The clinical use of CysC has in the past been limited by higher cost and the blood volume needed for analysis, which, however, is currently likely not a significant factor in many centers such as ours.

### CDH severity and risk of AKI

Our study reported a higher AKI incidence of 56–82% than the previously reported incidences of 37–63% in comparable CDH cohorts [18, 19, 23]. The higher incidence found in our study might be partially explained by the demographic and treatment differences between the reported cohorts. Our cohort includes a considerably high-risk population of neonates with CDH, with 16% of infants having undergone FETO, 57% with a higher-risk Type C/D defect, and 35% requiring ECMO, resulting in an overall mortality in our cohort of 18%. In comparison, only the cohort published by Arattu Thodika et al. [19], which consisted of 94 patients with an AKI incidence of 63%, included neonates with FETO (38%); the defect size was not reported; however, they had less frequent ECMO use (5%) and a higher mortality (37%). Liberio et al. [18] reported an AKI incidence of 38% in 90 neonates with CDH, with an unreported defect size and FETO use, but a high ECMO rate (36%), and comparable mortality (21%). The smallest CDH cohort, reported by Ryan et al. [23], consisted of 54 neonates with an AKI incidence of 37%; defect size and FETO use were not reported; however, ECMO use (35%) was similar to our cohort, with a slightly higher mortality of 31%. Gadepalli et al. [20] reported an AKI incidence of 77% in a sub cohort of 68 neonates with CDH requiring ECMO, with a 46% overall mortality rate, and a significant association between AKI and mortality. Other previous studies have used different definitions of kidney complications, such as the need for KRT, which was 35% in a cohort published by Tiruvoipati et al. [22], consisting of 165 neonates with CDH with 41% ECMO use and a 25% mortality. Rozmiarek et al. [21] reported kidney failure without further definition to be independently associated with mortality in a cohort of 111 neonates with CDH. Our study demonstrated o/e LHR, ECMO, and clinical sepsis as independent risk factors for AKI. Infants with a higher CDH severity therefore have a multifactorial increased risk of developing AKI, as they are likely to have lower o/e LHR, which puts them at higher risk for ECMO, and the installation of central lines and cannulas perpetuates a significant risk for sepsis [32].

### Neonates with CDH are exposed to a variety of risk factors for AKI

AKI can be caused by a variety of factors, including infection or inflammation, hypoperfusion, and hypoxia of the kidneys. Clinical sepsis was identified as an independent factor associated with AKI in our cohort, which may likely reflect the dual insult of systemic inflammation and hemodynamic instability [33, 34]. In addition, treatment of sepsis, especially central-line associated sepsis, requires the use of potentially nephrotoxic drugs, such as vancomycin. In our cohort, vancomycin administration and duration were significantly associated with AKI in univariate analysis, underscoring the importance of quality improvement strategies to primarily prevent infection whenever possible [32, 35]. Our study also demonstrated that CDH patch repair was associated with evidence of AKI in univariate analysis. Although patch repair did not emerge as an independent risk factor in our cohort and may be secondary to overall defect size and CDH severity, an interesting study published by Morozov et al. [36] measured intraabdominal pressures by intravesical manometry and kidney doppler ultrasound after CDH repair in 27 newborns, of which 13 had CDH. They demonstrated significantly elevated intraabdominal pressures with consecutively reduced kidney perfusion after CDH repair, with increased biomarkers of kidney hypoxic injury. Abdominal surgery, patch repair, and increased intraabdominal volume, which may be secondary to third spacing in the context of sepsis or postoperative systemic inflammation, have all been shown to be risk factors for intraabdominal hypertension with consecutive kidney dysfunction and AKI [37]. In our cohort, depending on AKI definition, 56–82% of ECMO patients developed AKI, which is similar to the 77% incidence of AKI among ECMO patients reported by Liberio et al. [18]. Another potential explanation for the higher incidences observed in our cohort is the relatively high frequency of laboratory measurements of SCr and CysC per patient (median CysC samples per patient, 2 [0–9]; median SCr samples, 15 [1, 9–21]). However, the frequency of SCr and CysC measurements in the other cohorts remains unclear.

In our unit, we do not routinely use NIRS (near-infrared spectroscopy) as an indicator of kidney hypoxia, or routinely measure kidney vascular perfusion with ultrasound, so we are unable to correlate our findings with these potential causes of AKI in this study. Neonates with CDH are prone to inflammation, which may be associated with endothelial dysfunction and pulmonary hypertension, and our group has recently demonstrated that the proinflammatory response may be attenuated in neonates with CDH receiving ECMO and is associated with increased mortality [38]. The interplay between extracorporeal support and AKI is likely multifactorial, as kidney dysfunction may be caused by multiple factors such as hemolysis, inflammation, altered perfusion, and hypoxia, which may also influence each other [39]. Future prospective studies should aim to follow protocols that combine NIRS, ultrasound, and laboratory markers to distinguish the occurrence, influence, and timeline of hypoxia, hypoperfusion, and inflammation as potential causes of AKI in neonates with CDH.

### Impact of AKI and diagnostic challenges

In clinical practice, the diagnosis of AKI remains a challenge, as the use of accurate glomerular filtration rate (GFR) as measured by inulin clearance as the gold standard is not practical and thus unsuitable for routine use. Therefore, different guidelines—using a trend in different markers rather than estimated GFR (eGFR)—exist to diagnose AKI, using either SCr, urine output, or more recently CysC. Furthermore, CysC and SCr are more secondary mirrors of reduction in glomerular filtration and not biomarkers of direct cellular injury like NGAL. In our study, we used only SCr and CysC to assess pRIFLE, nKDIGO, and CyNA for diagnosing AKI, and not eGFR or urine output measurements. The use of SCr alone may underestimate AKI; however, one recently published study by Andersson et al. [40] demonstrated that adding urine output did not identify any additional cases of AKI in a cohort of 109 neonates with CDH, with an AKI incidence of 28%, concluding that AKI in CDH neonates may be mainly non-oliguric. We did not include urine output in our study, as data were only available for 40% of the neonates in our cohort, and the accuracy of measurements may be influenced by the use of diuretics, or practical circumstances, such as inaccurate measurements of diapers or leaking foley catheters among other confounding factors. Andersson et al. [40] demonstrated a lower incidence of AKI (28%) as defined by nKDIGO in their cohort; interestingly, however, they observed a high incidence of mild (55%) and severe (42%) AKI in their cohort, but only 3% of patients had moderate AKI. Severe AKI primarily occurred in patients requiring ECMO, and 42% of all AKI patients received KRT. Aside from the demographic and clinical differences, one of the main differences between previously published cohorts and our study is that all of the above-mentioned studies used SCr-derived AKI definition criteria with or without urine output, whereas we also included the CysC-derived CyNA definition [10].

### Limitations

Our study has several limitations. The retrospective single-center design may cause bias, and causal relationships between individual risk factors and AKI cannot be established definitively. While the sample size may be inadequate to provide sufficient statistical power for some assessed variables and secondary outcomes, the number of included infants is the strength of our study, as we report the largest monocentric population of neonates with CDH investigating AKI to date. Our AKI criteria were only based on kidney dysfunction biomarkers, and did not include GFR, urine output data, or a clear kidney injury marker. Another limitation is that follow-up data beyond the NICU stay were not available, precluding assessment of post-discharge kidney or cardiovascular outcomes. Finally, this study did not systematically examine echocardiographic data of pulmonary hypertension and cardiac dysfunction in relation to AKI, which may be a valuable addition for future investigations considering the correlation between SCr/CysC and altered cardiac structure. To validate our findings and establish long-term implications and follow-up needs, prospective multicenter studies with standardized biomarker sampling and longitudinal cardiac and kidney follow-up would be required.

## Conclusions

AKI is a frequent and serious complication in neonates with CDH and is associated with longer hospitalization and mortality. Risk factors include overall disease severity, sepsis, and ECMO. CysC seems to be an appropriate biomarker for kidney dysfunction in neonates with CDH and may help identify more neonates with CDH who are at high risk of severe AKI. Overall, our findings indicate that SCr and CysC provide complementary information in the assessment of AKI in neonates with CDH rather than support the clear superiority of one biomarker over the other.

## Supplementary information

Below is the link to the electronic supplementary material.

Graphical abstract (PPTX 464 KB)

## Data Availability

The data that support the findings of this study are available upon request from the corresponding author. The data are not publicly available due to privacy or ethical restrictions.
