# Supplementary material for: Comparison of creatinine- and cystatin C–based definitions of acute kidney injury in neonates with congenital diaphragmatic hernia
Source: Pediatr Nephrol. 2026 Jan 25;41(7):2169–83. doi: 10.1007/s00467-026-07156-2 (PMC13197386; doi:10.1007/s00467-026-07156-2)
Supplement: Supplementary file 1 — Graphical abstract (PPTX 464 KB) [file 467_2026_7156_MOESM1_ESM.pptx]

## Slide 1
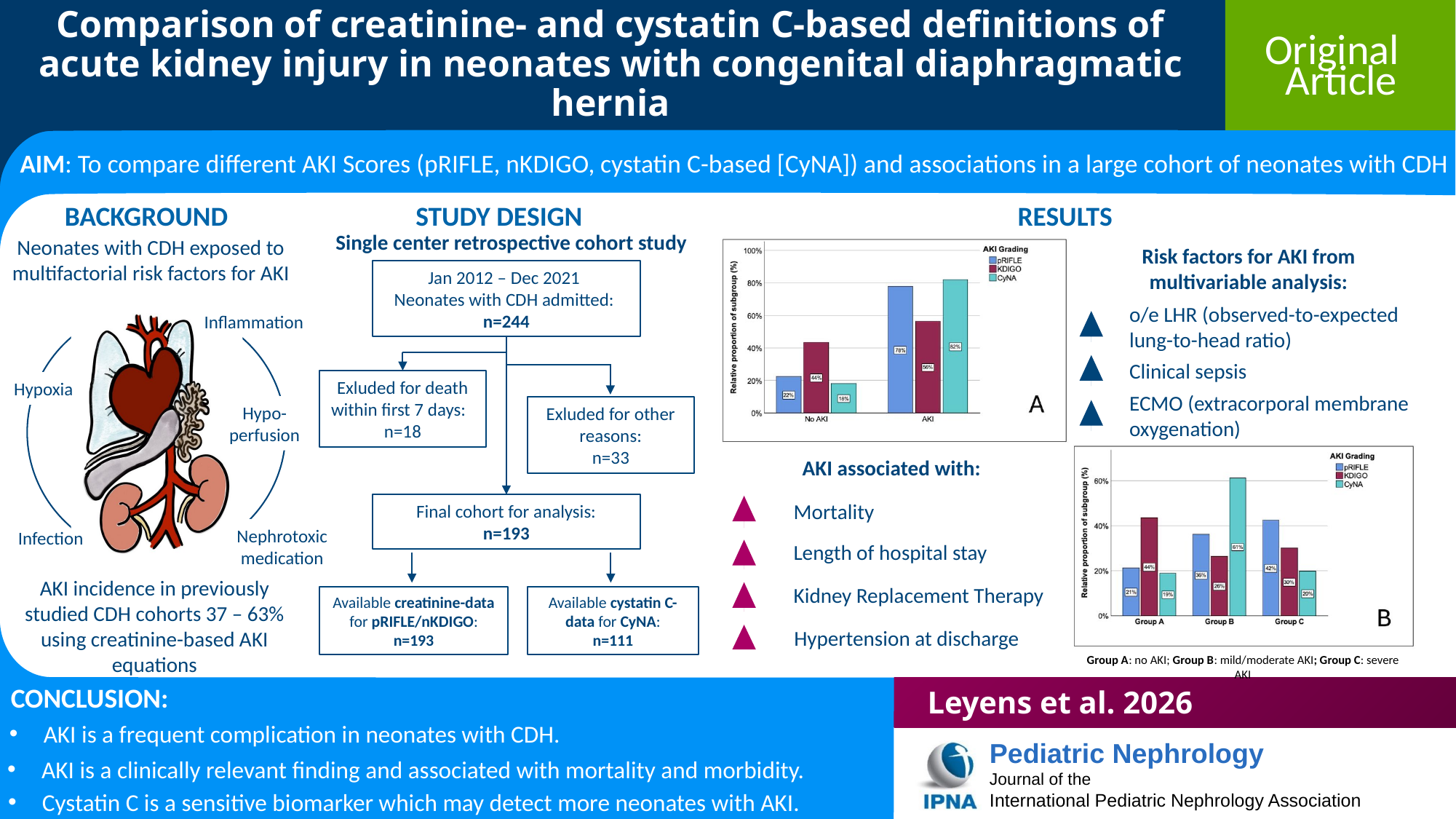

Comparison of creatinine- and cystatin C-based definitions of acute kidney injury in neonates with congenital diaphragmatic hernia
AIM: To compare different AKI Scores (pRIFLE, nKDIGO, cystatin C-based [CyNA]) and associations in a large cohort of neonates with CDH
BACKGROUND
STUDY DESIGN
RESULTS
Single center retrospective cohort study
Neonates with CDH exposed to multifactorial risk factors for AKI
Risk factors for AKI from multivariable analysis:
Jan 2012 – Dec 2021
Neonates with CDH admitted:
n=244
o/e LHR (observed-to-expected lung-to-head ratio)
Inflammation
Clinical sepsis
Exluded for death within first 7 days:
n=18
Hypoxia
ECMO (extracorporal membrane oxygenation)
Hypo-perfusion
Exluded for other reasons:
n=33
AKI associated with:
Mortality
Final cohort for analysis:
n=193
Nephrotoxic medication
Infection
Length of hospital stay
AKI incidence in previously studied CDH cohorts 37 – 63% using creatinine-based AKI equations
Kidney Replacement Therapy
Available creatinine-data for pRIFLE/nKDIGO:
n=193
Available cystatin C-data for CyNA:
n=111
Hypertension at discharge
Group A: no AKI; Group B: mild/moderate AKI; Group C: severe AKI
CONCLUSION:
Leyens et al. 2026
AKI is a frequent complication in neonates with CDH.
AKI is a clinically relevant finding and associated with mortality and morbidity.
Cystatin C is a sensitive biomarker which may detect more neonates with AKI.
